# The economic burden of Lyme disease and the cost-effectiveness of Lyme disease interventions: A scoping review

**DOI:** 10.1371/journal.pone.0210280

**Published:** 2019-01-04

**Authors:** Stephen Mac, Sara R. da Silva, Beate Sander

**Affiliations:** 1 Institute of Health Policy, Management and Evaluation, University of Toronto, Toronto, Ontario, Canada; 2 Toronto Health Economics and Technology Assessment (THETA) Collaborative, University Health Network, Toronto, Ontario, Canada; 3 Department of Biology, University of Toronto Mississauga, Mississauga, Ontario, Canada; 4 Institute for Clinical Evaluative Sciences, Toronto, Ontario, Canada; 5 Public Health Ontario, Toronto, Ontario, Canada; Kingston University, UNITED KINGDOM

## Abstract

**Introduction:**

While Lyme disease (LD) is mostly treatable, misdiagnosed or untreated LD can result in debilitating sequelae and excessive healthcare usage. The objective of this review was to characterize the body of literature on the economic burden of Lyme disease (LD) and the cost-effectiveness of LD interventions, such as antibiotic treatment and vaccination.

**Methods:**

We followed Joanna Briggs Institute scoping review methodologies. We systematically searched terms related to LD, economic evaluations, costs, and cost-effectiveness in Medline, Embase, PsycInfo, Cochrane Library, and the grey literature up to November 2017. We included primary economic evaluations conducted in North America and Europe, reporting LD-related costs or cost-effectiveness of human interventions. Two reviewers screened articles and charted data independently. Costs were standardized to 2017 United States dollars (USD).

**Results:**

We screened 923 articles, and included 10 cost-effectiveness analyses (CEA) and 11 cost analyses (CA). Three CEAs concluded LD vaccination was likely cost-effective only in endemic areas (probability of infection ≥1%). However, LD vaccination is not currently available as an intervention in the US or Europe. Six studies assessed economic burden from a societal perspective and estimated significant annual national economic impact of: 735,550 USD for Scotland (0.14 USD per capita, population = 5.40M), 142,562 USD in Sweden (0.014 USD per capita, 9.96M), 40.88M USD in Germany (0.51 USD per capita, 80.59M), 23.12M USD in the Netherlands (1.36 USD per capita, 17.08M), and up to 786M USD in the US (2.41 USD per capita, 326.63M).

**Conclusions:**

Lyme disease imposes an economic burden that could be considered significant in the US and other developed countries to justify further research efforts in disease control and management. Societal costs for Lyme disease can be equally impactful as healthcare costs, but are not fully understood. Economic literature from countries with historically high incidence rates or increasing rates of Lyme disease are limited, and can be useful for future justification of resource allocation.

## Introduction

Lyme disease (LD), also known as Lyme borreliosis, is an increasingly common vector-borne disease reported in temperate climate zones in North America (NA) and parts of Europe.[1–3] Most human LD infections are caused by three species of bacteria: *Borrelia burgdorferi*, *B*. *garinii*, *and B*. *afzelii* [1] Since 2015, LD has been the most common reportable vector-borne disease in NA and Europe.[3,4] Endemic areas in Europe (e.g. Slovenia) and the United States (US) (e.g. Maine) have reported incidence rates of 130 per 100,000 populations in 2010, and 86.4 per 100,000 populations in 2016, respectively. [3,4] Furthermore, current reported rates of LD may be conservative given underreporting estimates of eight to tenfold in the United States. [1] The World Health Organization has made LD a priority disease,[5] as experts predict escalating climate change to play a significant role in the proliferation of this disease due to the expansion of habitable environments for ticks.[6] In Canada, the controversies surrounding the clinical management of LD prompted the federal government to commit to addressing the challenges of recognition, timely diagnosis and treatment of LD, mandated by the unprecedented Federal Framework on Lyme Disease Act.[7]

While mostly treatable, misdiagnosed or untreated LD can result in debilitating long-term sequelae, inappropriate long-term antibiotic therapy and excessive healthcare use.[8] There is currently no human LD vaccine available.[9] The objective of this review was to systematically gather and characterize the body of literature on the economic burden of LD and the cost-effectiveness of LD intervention strategies in order to identify possible knowledge gaps affecting health policy decision-making for LD.

## Methods

This scoping review followed the five-step framework by Arksey and O’Malley with guidance from the Joanna Briggs Institute.[10,11] PRISMA (Preferred Reporting Items for Systematic Reviews and Meta-Analyses) guidelines were followed.[12]

### Search strategy

A scientific literature search was conducted for English language studies published in four electronic databases from inception to November 2017: Medline In-Process and Other Non-Indexed Citations database (Ovid interface), Embase (Ovid interface), PsycInfo (Ovid interface) and the Cochrane Library (Cochrane Central Register of Controlled Trials), Cochrane Database of Systematic Reviews, Health Technology Assessment (HTA) Database, NHS Economic Evaluation Database and Database of Abstracts of Reviews of Effects). Search terms were developed in consultation with a faculty librarian at the University of Toronto Libraries and included the concepts: “Lyme disease”, “Lyme borreliosis”, “healthcare costs”, “health economics”, “cost-effectiveness analysis”, “economic evaluations”, “*Borrelia* infections”, and LD stages or manifestations such as: “erythema chronicum migrans”, “Lyme neuroborreliosis” and “post-treatment Lyme disease”. The complete Medline search strategy is presented in S1 Text. This strategy was adapted for use in other databases to adjust for database-specific syntax.

### Searching other sources

Reference lists from relevant articles and systematic reviews were manually searched to identify further relevant studies for potential inclusion. Grey literature was searched following the Canadian Agency for Drugs and Technology in Health (CADTH) guidelines.[13] A total of 48 HTA agencies and health economic organizations in NA and Europe were searched using concepts similar to the electronic database searches.

### Eligibility criteria

We included the following eligible economic evaluations: cost-of-illness analysis, cost-minimization analysis, cost-effectiveness analysis, cost-utility analysis, and cost-benefit analysis. For analysis, we classified studies as a CEA if it included a cost, health and cost-effectiveness outcome (e.g. cost per case averted, or cost per quality-adjusted life year (QALY) gained). We classified economic evaluations as cost analyses if the outcomes were solely focused on costs (e.g. diagnostic, total healthcare, treatment) and if the study was comprehensively conducted using the literature or real-world data.[14] Studies reporting a simple cost estimate and/or referencing a primary study were excluded. CEA studies that did not evaluate LD-associated interventions for humans were also excluded.

Due to the comparable health care systems and the nature of LD, we included studies conducted in NA (Canada and US) and Europe (all 51 countries). There were no limitations on the publication date and we searched up until November 8^th^, 2017. Editorials, reviews, comments, replies, correspondences, viewpoints and protocols were excluded. Articles that reported outcomes unrelated to costs, health outcomes and/or economic evaluation outcomes were excluded.

### Study selection

**Fig 1:**
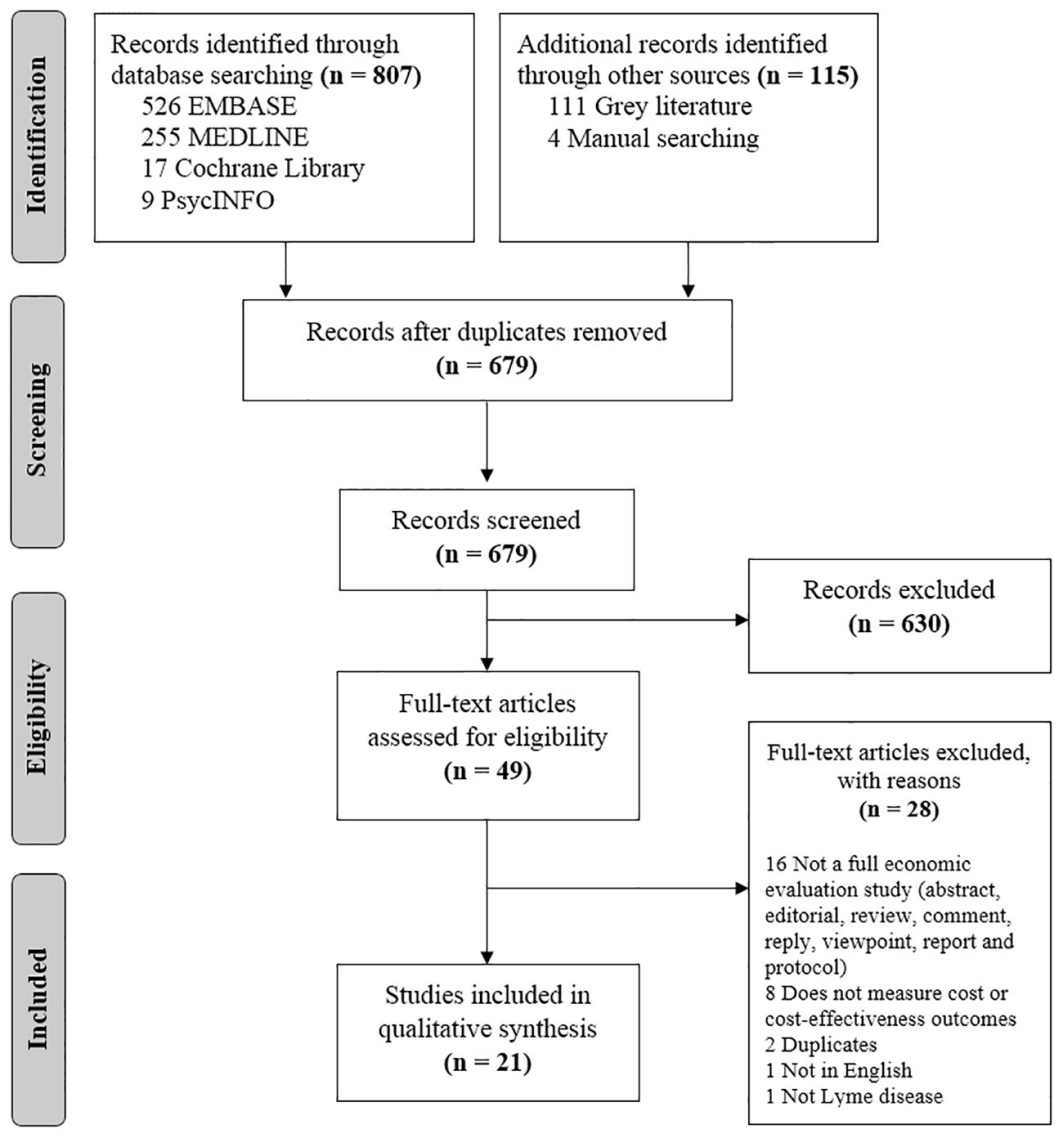
Literature search and study selection.

All search results were aggregated and de-duplicated using Mendeley Reference Management Software. Abstract and title, and full-text screening were completed independently by two reviewers (SM and SDS). Prior to screening, both reviewers conducted a calibration with a set of 100 results. Conflicts at any stage of screening were discussed and resolved through consensus. Disagreements were resolved by a third reviewer (BS). Study selection process and exclusion reasons are shown in Fig 1.

### Data charting

Data was extracted independently in duplicate (SM and SDS). Data extracted included: authors, publication year, country where study was conducted, economic evaluation type, study objective, data sources, outcomes reported, model type (CEA) or analytical technique (cost analyses), strategies compared (CEA), study perspective, time horizon, use of sensitivity analysis, discounting, use of a cost-effectiveness threshold (CET), and study findings. Since the objective of this Review was to summarize the existing literature, as well as to identify knowledge gaps in LD economic evidence, protocol registration, quality appraisal and meta-analyses were not conducted.

### Summarizing results

A descriptive analysis was used to summarize studies included in the review. Themes for analysis include the type of economic evaluation conducted, countries/ regions where the study was conducted, types of outcomes reported, and the use of economic evaluation concepts recommended by the Consolidated Health Economic Evaluation Reporting Standards (CHEERS) statement.[15] A descriptive analysis of the interventions compared in CEA economic evaluations, and the types of costs in costing economic evaluations were summarized. Costs were inflated to 2017 local currencies and standardized to US dollars (USD). Economic burden was expressed in cost per capita of the respective countries.[16] Results were stratified into pre-2003 and post-2003 periods to explore any trends resulting from the withdrawal of human LD vaccine in February 2002. [9]

## Results

### Literature search

Systematic searches resulted in a total of 923 records. After screening, a total of 21 studies were included in the final analysis (Fig 1), 20 of which were peer-reviewed manuscripts and one report. Ten studies were categorized as CEA,[17–26] and 11 were categorized as cost analyses.[27,28,37,29–36]

### Descriptive analysis of economic evaluations

**Fig 2:**
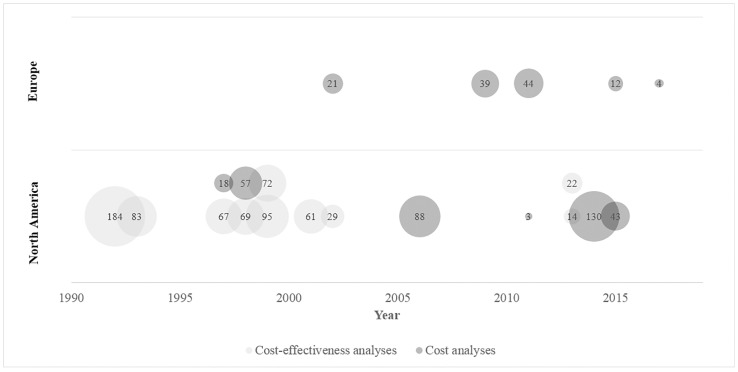
Bubble chart displaying studies included in review by study year, geographic region, and type of economic evaluation. Balloon size depicts the number of citations through Google Scholar.

Fig 2 presents an overview of studies categorized by economic evaluation type, geographic region of origin, publication year and impact based on number of Google Scholar citations. The majority of included economic evaluations (n = 11, 52%) were published prior to 2003, with eight CEA[17–24] and three cost analyses.[27–29] From 2003 and onwards, there were 10 published economic evaluations: two CEA, [25,26] and eight cost analyses. [30–37] All LD intervention CEAs were from NA countries, while cost analyses were published more frequently after 2003, and from European countries. The cost-effectiveness of antibiotic treatment strategies,[17] and a diagnostic test cost analysis,[34] were considered most impactful studies based on their number of Google Scholar citations.

### Data source usage

**Fig 3:**
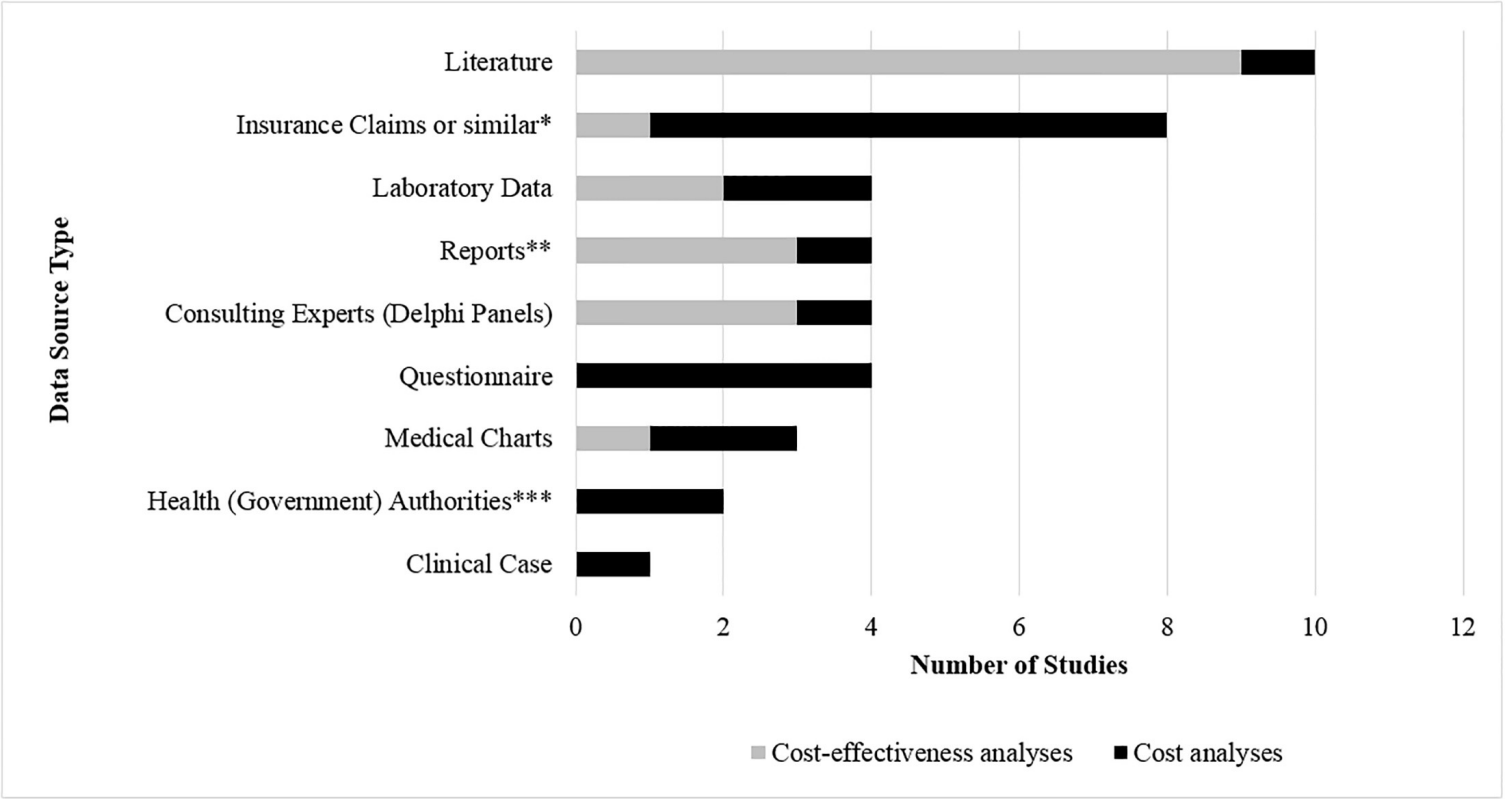
Data sources used by LD cost-effectiveness analyses and cost analyses. * New Jersey Blue Cross—Blue Shield; Diversified Pharmaceutical Services; Delmarva Health Plan; Swedish Social Insurance; German DAK; IMS Health LifeLink Health Plan Claims; ** CDC LD Incidence Reports, Epidemiologic Reports, Lyme Disease Vaccine Study Group; *** Scottish Health Service, Departments of Economy and Information Technology.

Data sources used by the 21 economic evaluations, stratified by CEA and cost analysis, were summarized in Fig 3. Literature use was most common (n = 10, 48%), followed by insurance claims information (n = 8, 38%). CEAs mostly used existing literature, reports, and consulting experts, while cost analyses mostly used insurance claims or health provider data as well as questionnaire data to complete their economic evaluations. There were no economic evaluations that used health administrative data from a single payer health system.

### Economic evaluations assessing cost-effectiveness

#### Study characteristics

Study design characteristics are summarized in Table 1. Six CEA studies used a healthcare payer perspective, [17–20,24,25] and four studies used a societal perspective,[21–23,26] of which three compared the cost-effectiveness of a vaccination program.[21–23] One study also used a hospital perspective in the sensitivity analysis.[26]

**Table 1:** Economic evaluation study characteristics.

Study Characteristics, n (%)	CEA (n = 10)	Cost analysis (n = 11)
**Year of Study**		
Pre—2003	8 (80)	3 (27)
2003–2017	2 (25)	8 (73)
**Country where study conducted**		
North American countries	10 (100)	3 (27)
European countries^1^	0	8 (73)
**Number of strategies compared**		
Two or less	6 (60)	N/Ap
Three or more	4 (40)	N/Ap
**Type of CEA Model**		
Decision analysis	7 (70)	N/Ap
Markov cohort model	3 (30)	N/Ap
**Type of Costing Study**		
Healthcare costs	N/Ap	7 (64)
Diagnostic testing costs only	N/Ap	3 (27)
Treatment costs only	N/Ap	1 (9)
**Number of different outcomes included**		
Two or less	1 (10)	10 (91)
Three or more	9 (90)	1 (9)
**Study Perspective**^2^		
Healthcare payer	6 (60)	5 (45)
Societal	4 (40)	6 (55)
Other (hospital or third party payer)	1 (10)	2 (18)
**Time Horizon**		
0–5 years	4 (40)	10 (91)
5–10 years	3 (30)	-
> 10 years but not lifetime	1 (12)	-
Lifetime	2 (25)	1 (9)
**Use of Sensitivity Analysis**	9 (90)	4 (36)
**Use of Discounting**	6 (60)	2 (18)

^1^ Albania, Andorra, Armenia, Austria, Azerbaijan, Belarus, Belgium, Bosnia and Herzegovina, Bulgaria, Croatia, Cyprus, Czech Republic, Denmark, Estonia, Finland, France, Georgia, Germany, Greece, Hungary, Iceland, Ireland, Italy, Kazakhstan, Kosovo, Latvia, Liechtenstein, Lithuania, Luxembourg, Macedonia, Malta, Moldova, Monaco, Montenegro, Netherlands, Norway, Poland, Portugal, Romania, Russia, San Marino, Serbia, Slovakia, Slovenia, Spain, Sweden, Switzerland, Turkey, Ukraine, United Kingdom, Vatican City

^2^ Perspectives were classified based on the costs included by the authors if it was not explicitly stated; percentages may not add up to 100% in certain cases if studies examined multiple perspectives.

**CEA**, Cost-effectiveness analysis; **N/Ap**, Not applicable

The time horizon ranged between one-year and lifetime, with seven studies using a time horizon less than 10 years, [17,18,22–26] and only two using a lifetime horizon.[19,20] Ninety percent of CEAs completed a deterministic sensitivity analysis.[17–25] Probabilistic sensitivity analyses were not presented. Discounting was used in six studies, [19–24] where rates varied between 3% and 5% for base-case analyses, and between 0% (i.e. no discounting) and 10% in sensitivity analyses.

#### Model type and interventions assessed

Seven CEAs reported an expected cost-effectiveness outcome using decision tree analysis [17,18,20–22,25,26];three CEAs used a Markov state-transition model.[19,23,24]. Four studies assessed the cost-effectiveness of vaccination.[21–24] Five CEAs assessed antibiotic cost-effectiveness: three studies compared treatment algorithms for early and late disseminated sequelae, [17,19,20] one compared treatment algorithms for early localized LD, [25] and one assessed cost-effectiveness of intravenous ceftriaxone in patients who lacked classical clinical manifestations.[18]

#### Outcomes

Cost, health, and cost-effectiveness outcomes reported are summarized in Table 2. Types of direct costs (healthcare costs) incorporated into models included: laboratory diagnostics, physician services, hospital care, medication and associated-adverse events (e.g. antibiotic treatment), sequelae, vaccination (e.g. administration, time, travel), and cost per LD case. Indirect costs included productivity loss.

**Table 2:** Primary study characteristics and conclusions of cost-effectiveness analyses.

Author	Country	Outcomes	Comparator Strategies	Model Type	Conclusions (Unadjusted)
Magid et al. [17]	USA	**1. Costs**: lab tests, physician services, hospital care, and hospital medications **2. Health Outcomes**: Major and minor complications (sequelae), patients reactions to antibiotics **3. ICER**: USD per additional major complications prevented	**1. Treat all**, Empirically treat all patients with 2 weeks of doxycycline **2. Follow**, Treat only patients in whom EM develops **3. Test**, Treat only patients with EM or a positive serologic test for LD one month after exposure	Decision analysis	Empirical treatment (“Treat All”) of patients with tick bites was most cost-effective when the probability of infection after a bite is ≥ 0.036 or higher. If probability of infection < 0.01, “Follow” is most cost-effective compared to other strategies.
Lightfoot et al. [18]	USA	**1. Costs**: antibiotic treatment, side effects **2. Health outcomes**: side effects of ceftriaxone therapy, getting late LD **3. ICER**: USD per case of late LD prevented	**1. Empirical IV antibiotic treatment**, of non-specifically symptomatically patient who has a positive Lyme antibody titer **2. No antibiotic treatment**	Decision analysis	Empirical treatment resulted in an ICER (USD 1993) of $86,221 per LD case prevented. For most patients with a positive Lyme antibody titer and non-specific symptoms, the risks and costs of empirical parenteral antibiotic therapy exceed the benefits (i.e. not cost-effective)
Eckman et al.[19]	USA	**1. Costs**: hospitalization for complications of antibiotic therapy, ambulatory visits, and treatment **2. Health outcomes**: Anaphylaxis, minor and major complications, quality of life **3. ICER**: USD per QALY	**1. Oral therapy with 100 mg of doxycycline**, twice daily for three weeks (in patients with early LD) or four weeks (in those with Lyme arthritis) 2. **At-home IV administration of ceftriaxone**, 2 grams once a day for two weeks (in patients with early LD) or four weeks (in those with Lyme arthritis)	Markov cohort model	When compared to IV ceftriaxone for treatment of early LD and Lyme arthritis, oral therapy of doxycycline was dominant (i.e. cost savings of $544 and $546, and health benefits of 0.1 QALY for both early LD and Lyme arthritis, respectively).
Nichol et al.[20]	Canada	**1. Costs**: Testing, therapy for each syndrome, treatment, minor/major side effects, sequelae **2. Utilities**: Time Trade-off **3. Health Outcomes**: Life expectancy, QALY, sequelae **4. ICER**: USD per QALY	**1. No testing-no treatment** **2. Testing**, with enzyme-linked immunosorbent assay (ELISA) followed by antibiotic treatment of patients with positive results **3. Two-step testing**, with ELISA followed by Western blot and antibiotic treatment for patients with positive results on either test **4. Empirical antibiotic therapy**	Decision analysis	For myalgic symptom patients, the “no testing-no treatment” strategy was most economically attractive. For patients with EM-resembling rash, “Treat All” was the most cost-effective strategy. For patients with oligoarticular arthritis, the “two-step testing” was most economically attractive. Empirical treatment is most attractive when the annual incidence of new infection or pretest probability of LD was high.
Meltzer et al. [21]	USA	**1. Costs**: vaccination costs (administration, time, travel, AE), cost of treating a case of LD **2. Health Outcomes**: LD cases averted, sequelae due to early or late, disseminated infection, cases resolved **3. ICER**: USD per case averted	**1. Vaccination** **2. No vaccination**	Decision analysis	The ICER (USD 1999) for vaccination was 4.466 USD per LD case averted. Vaccination was not considered cost-effective for universal use. Economic benefits are greatest when the probability of contracting LD > 0.01.
Stratton et al. [22]	USA	**1. Costs**: diagnostic evaluation, physician visits, and antibiotic treatment (assumed two visits and prescription antibiotic and half patients get a diagnostic test), vaccination development costs **2. Health Outcomes**: total deaths, total cases, life expectancy, utilities, QALY **3. ICER**: USD per QALY	**1. Vaccination**, including development **2. No vaccination**	Decision analysis	The ICER (USD 1999) was $3.5M per QALY if a vaccine program were developed and implemented assuming 100% efficacy and 100% utilization by the target population. Vaccine candidate for LD was not considered cost-effective.
Shadick et al. [23]	USA	**1. Costs**: Direct medical costs (management and treatment of LD, 3-shot vaccination series, adverse effects of vaccination, cost of antibiotic treatment), **1b. Indirect costs** (included in SA) **2. Health Outcomes**: Number of cases of LD, HRQoL, QALY **3. ICER**: USD per QALY	**1. Vaccination** **2. No vaccination**	Markov cohort model	At an LD incidence rate of 0.01, the ICER (USD 1998) was $62,300 per QALY and $5,300 per LD case averted. Vaccination appears only to be economically attractive for individuals who have a seasonal probability of *B*.*burgdorferi* infection of greater than 1%.
Hsia et al. [24]	USA	**1. Costs**: vaccine/booster and administration, antibiotics, adverse drug reactions, major and minor sequelae **2. Health Outcomes**: cases averted **3. ICER**: USD per case averted	**1. Vaccination** **2. No vaccination**	Markov cohort model	At an LD incidence rate of 0.01, the ICER (USD 1999) was $9,900 per LD case averted. At average national incidence rate of 0.0067%, the ICER was $1.6M per case averted. Vaccination is not cost-effective for universal use in the US; only for individuals who live endemic areas.
Lantos et al. [25]	USA	**1. Costs**: antibiotic treatment, laboratory testing, disseminated LD, major adverse medication effects, sequelae, and serology **2. Health outcomes**: Cases averted per 100,000 patients, Disseminated cases per 100,000 patients, major AE per 100,000 patients **3. ICER**: USD per patient, USD per case averted	**1. Treat All**, all patients given a standard course of antibiotics intended to treat EM due to early LD **2. Observe**, treat only if disseminated LD developed **3. Serology**, patients are tested using standard two-tier serology (ELISA followed by WB) and antibiotics are given to those meeting criteria for seropositivity, while negative test patients are observed	Decision analysis	All strategies became more costly as the P (LD | EM) increased. In terms of costs per patients, “Treat All” was cost-effective compared to the other strategies when P (LD | EM). > 0.0061. In costs per averted disseminated LD, “Treat All” was always cost-effective when compared to the “Serology” strategy regardless of P (LD | EM).
Wormser et al. [26]	USA	**1. Costs**: Median direct costs (undiscounted) of LD serological tests, and median net costs (reimbursement reflect median Centers for Medicare and Medicaid Services for 2012) **2. Outcomes**: Test sensitivity and specificity **3. ICER**: None	**1–2**. The C6 Lyme ELISA kit plus either of two WCS ELISAs **3–4**. The immunoblot assays used with the Lyme IgG and IgM immunoblot kits from MarDx/Trinity Biotech	Decision analysis	The WCS ELISA followed by the C6 ELISA was a dominant testing strategy (i.e. cost saving by 27.1% to 44%, and more sensitive).

**AE**, Adverse events; **CEA**, Cost-effectiveness analysis; **EE**, Economic evaluation; **EIA**, Enzyme immunoassay test; **ELISA**, Enzyme-linked immunosorbent assay; **EM**, Erythema migrans; **HRQoL**, Health-related quality of life; **ICER**, Incremental cost-effectiveness ratio; **IgG**, Immunoglobulin G; **IgM**, Immunoglobulin M; **IV**, intravenous; **LB**, Lyme borreliosis; **LD**, Lyme disease; **N/Ap**, Not applicable; **NR**, Not reported; **P (LD | EM)**, Probability of Lyme disease given erythema migrans rash; **QALY**, Quality-adjusted life years; **SA**, Sensitivity analysis; **USA**, United States of America; **USD**, United States dollar; **WCS**, whole cell sonicate

Health outcomes chosen for CEAs included: the number of major and minor complications (sequelae), number of therapy-related adverse events, number of LD cases averted, life expectancy, QALYs, and mortality. One study used test sensitivity and specificity outcomes. [26]

Incremental cost-effectiveness ratios (ICERs) reported were: cost per additional major complications prevented, cost per late LD case prevented, cost per QALY, and cost per LD case averted. One study did not conclude a cost-effectiveness ratio outcome.[26]

### Study findings

Conclusions from all CEA are summarized in Table 2. All ICERs were inflated and standardized to 2017 USD per QALY. The ICER for vaccination programs are summarized in Table 3 and ranged between 7,024 USD (probability of LD infection of 0.5%, [21]) and 2.36M USD (probability of LD infection of 0.0067%, [24]) per LD case averted. Studies reporting the ICER in USD per QALY reported results between 93,619 (probability of infection of 1%, [23]) and 5.17M (probability of infection of 0.0046%, [22]) USD per QALY. The ICER varied depending on the probability of LD infection, probability of diagnosing early LD and vaccination costs. These three CEAs, all from societal perspectives, concluded that vaccination was likely economically favorable for endemic LD areas and not cost-effective for nation-wide administration. All four studies used a time horizon of 10 to 11 years, performed sensitivity analyses, and discounted at 3%.

**Table 3:** Summary of standardized ICER for vaccination programs in the United States.

Probability of LD infection (Incidence rates) (%)	ICER (2017 USD per case)	ICER (2017 USD per QALY)	Reference
0.0046		5,170,000	[22]
0.0067	2,360,000		[24]
0.5	7,024		[21]
1	7,964	93,619	[23]
1	14,632		[24]

**ICER**, Incremental cost-effectiveness ratio; **LD**, Lyme disease; **QALY**, Quality-adjusted life year; **USD**, United States Dollar

In 1992, Majid *et al*. concluded that empirical antibiotic treatment of patients with tick bites was cost-effective when the probability of infection was 0.036 or higher.[17] Subsequent studies by Lightfoot *et al*., Nichols *et al*., and Lantos *et al*., also reported that an empirical antibiotic approach is cost-effective and preferred for patients with a positive Lyme antibody titer, if the pretest probability for LD is high, and for patients in regions endemic for LD.[18,20,25] The study by Eckman *et al*. assessed the cost-effectiveness of oral antibiotic treatment using 100 mg of doxycycline compared to an intravenous administration of 2 g of ceftriaxone. This study concluded that oral doxycycline was dominant (cost savings and provided an additional 0.1 QALY) in both early LD and Lyme arthritis patients.[19]

### Economic evaluations assessing LD-associated costs

#### Study characteristics

Seven studies assessed the economic burden of LD using total healthcare costs,[28–31,35–37] three studies included diagnostic testing costs only,[27,32,34] and one study included Lyme cardiac treatment costs only.[33] Five studies used a healthcare payer perspective,[27,32–35] six studies used a societal perspective, [28–31,36,37] and two studies used a third-party payer perspective.[28,36] Approximately 91% (10 of 11) of cost analyses used a time horizon between 0 and 5 years.[27–36] Only four studies completed any form of sensitivity analysis, [29,33,35,37] and two studies used discounting with rates between 3 and 4%.[28,37]

#### Outcomes

Outcomes reported are summarized in Table 4. Cost analyses focusing on overall healthcare costs included direct medical costs: outpatient visits and related healthcare utilization, hospitalizations, emergency room visits, home health care, prescription medication (antibiotic treatment), cost of subsequent manifestations (major or minor sequelae), consultations, laboratory costs, and treatment side effects. Diagnostic cost analyses only included serologic test costs and laboratory costs.

**Table 4:** Primary study characteristics and conclusions of cost analyses.

Authors; Year	Country	Cost Type; Perspective	Outcomes	Conclusions (Unadjusted Costs)
Strickland et al.; 1997 [27]	USA	Diagnostic; Healthcare payer	**1. Costs**: serologic tests (EIA and Western Blot)	Physicians in Maryland often used EIAs to follow patients after treatment, an inappropriate practice that increases the overall cost of testing for LD. A total of 30,000 tests for LD were performed annually in Maryland adding an annual burden of $3.23 million ($2 million, USD 1995) in direct medical costs.
Maes et al.; 1998 [28]	USA	Healthcare; Societal	**1. Direct Costs**: Direct medical costs (outpatient visits, hospitalizations, emergency room visits, home health care, and prescription medication, cost per episode, cost of chronic manifestations); **1b. Indirect Costs**: work loss, restricted-activity days at home) **2. Health Outcomes**: Stage II and III sequelae prevented	Using an annual mean incidence of 4.73 cases of Lyme disease per 100,000 population, the model extrapolated expenditures from US endemic areas and yielded an expected national expenditure of $3.93 billion ($2.5 billion, USD 1996) over 5 years for therapeutic interventions to prevent 55,626 cases of Lyme disease sequelae. This study suggested the need to develop vaccination strategies for specific target groups.
Joss et al.; 2002 [29]	Scotland	Healthcare; Societal	**1. Direct Costs**: Direct costs of consultation; laboratory costs; antibiotic treatment, including a percentage increment for possible major or minor side-effects; cardiac, neurologic or musculoskeletal/ arthritic sequelae); **1b. Indirect Costs**: loss of healthy time, and from sequelae **1c. Costs, probable cases**: consultation and screening tests costs	From a societal perspective, the total annual national economic burden of LD in Scotland was estimated to be £543,678 (£331,000, range £47,000–615,000, Sterling Pound 1999). An additional annual cost of £125,000–£156,513 (£76,000 –£95,000, Sterling Pound 1999) was spent for patients with a concern and no certainty of contracting LD. These costs were not included in the national estimate.
Zhang et al.; 2006 [30]	USA	Healthcare; Societal	**1. Direct Medical Costs**: LD diagnosis and treatment, physician visits, consultation, serology, procedure, therapy, hospitalization/ ER visits **1b. Indirect Medical Costs**: extra prescription and non-prescription drug costs paid out of pockets **1c. Non-medical Costs**: home or health aides, travel and caregiving **1d. Costs, productivity loss**: patient reported time lost from work (intangible costs of pain and suffering were not incorporated).	Additional direct medical costs and indirect medical costs were estimated at $4,273 ($2,970, USD 2000), and $7,484 ($5,202, USD 2000) respectively for early and or late stage LD patients. From a societal perspective, the annual national economic burden was estimated at $292M ($203M, USD 2000). Study concluded the need on further research on social behaviour and economic evaluations of LD prevention interventions.
Henningsson et al.; 2009 [31]	Sweden	Healthcare; Societal	**1. Direct Costs**: physician visits to outpatient department, hospitalization, antibiotic treatment **1b. Indirect Costs**: sickness benefit (temporary parental benefits) **2. Health Outcomes**: recovery after antibiotic treatment (full or partial)	From a societal perspective, the national economic burden of NB-related healthcare for Sweden over 5 years was estimated to be 598,119 EUR (500,000 EUR, EUR 2005) for the entire study group 3,948 EUR per patient (3,300 EUR), and the cost of social benefits was estimated to be 160,296 EUR (134,000 EUR), which is approximately 2,393 EUR (2,000 EUR) per patient. The study concluded that earlier diagnosis of borreliosis would result in reduced human suffering and in economic gain.
Muller et al.; 2011 [32]	Germany	Diagnostic; Healthcare payer	**1. Costs**: diagnostic testing, laboratory costs, treatment (separately)	In Germany, the overall expected burden from diagnostics was estimated at 57.0M EUR (51.2M EUR, EUR 2008) using diagnostic claims code data. The study’s conclusion suggested a high amount of potentially inappropriate healthcare services in patients with a suspected or confirmed diagnosis of LB.
Kim et al.; 2011[33]	USA	Treatment; Healthcare payer	1. **Costs**: PPM/EPM/TPW placement, hospital room and board, inpatient care in the Coronary Care Unit	At two weeks, the PPM cost $43,220 ($39,195, USD 2011) compared to EPM cost of $72,646 ($65,880) and TPW’s cost of $130,537 ($118,380) for Lyme conduction treatment. Significant cost savings can be realized if a PPM were initially implanted.
Hinckley et al.; 2014 [34]	USA	Diagnostic; Healthcare payer	**1. Costs**: Amount charged by commercial laboratories that is ultimately paid by insurance companies, Medicare/Medicaid, the patient, and/or the ordering medical center (e.g. hospitals, clinics)	Approximately 3.4M LD diagnostic tests were conducted by participating laboratories in 2008, at an estimated cost of $556M ($492M, USD 2008). LD testing was common and costly, even when testing was in accordance with diagnostic recommendations. It is important to consider clinical and exposure history in conjunction with diagnostic evidence.
Adrion et al.; 2015 [35]	USA	Healthcare; Healthcare payer	**1. Costs**: total inpatient, total pharmacy, total outpatient, outpatient anesthesiology, outpatient evaluation and management, outpatient medicine, outpatient pathology laboratory, outpatient radiology, outpatient surgery, and all other outpatient costs **2. Utilization**: outpatient management and evaluation visits, and emergency department visits	LD was associated with an increase of $3,048 ($2,968, 95% CI: 2,807–3,128, USD 2015) health care costs over a 12-month period. PTLDS-related diagnosis was associated with an increase of $3,946 ($3,798, 95% CI: 3,542–4,055) health care costs over a 12-month period, relative to those with no PTLDS related diagnoses. Using estimated costs, annual total medical costs attributable to LD and PTLDS could be between $740M and $1.35B ($712M and $1.3B) annually in the US.
Lohr et al.; 2015 [36]	Germany	Healthcare; Societal	**1. Direct Costs**: medical hospitalization costs **1b. Indirect Costs**: resulting from loss of productivity (human capital approach)	From a societal perspective, the annual national economic burden of LD in Germany was 34.3M EUR (30.8M EUR, EUR 2008) where the breakdown was 25.6M (23M) EUR for direct medical costs and 7.8M (7M) EUR for indirect costs. Study results were considered to be underestimated.
van den Wijngaard et al.; 2017 [37]	Netherlands	Healthcare; Societal	**1. Direct Costs**: GP consultations, specialist consultations, hospitalization, prescribed medications and formal home care **1b. Indirect costs, out of pocket**: informal care, self-paid household assistance, caregiving, OTC medication excluded **1c. Indirect Costs, production loss**: friction cost method and friction period of 12.1 weeks for work absenteeism **2. Health Outcomes**: no infection, asymptomatic infection, EM, disseminated LB, Lyme-related persisting symptoms	From a societal perspective, the annual national economic burden of LD in the Netherlands was estimated at 19.4M EUR (19.3M EUR, 95% CI 15.6–23.4, EUR 2014). Healthcare cost and production loss each constituted 48% of the total cost at 9.33M (9.3M) EUR and 9.23M (9.2M) EUR, respectively), while patient costs contributed 4% at 0.8M (0.8M) EUR. LB leads to a substantial societal cost. Further research should therefore focus on additional preventive interventions.

**ACER**, Average cost-effectiveness ratio; **AE**, Adverse events; **CI**, confidence interval; **EE**, Economic evaluation; **EIA**, Enzyme immunoassay test; **EM**, Erythema migrans; **EPM**, Externalized pacemaker; **EUR**, Euros; **GP**; General practitioner; **HRQoL**, Health-related quality of life; **LB**, Lyme borreliosis; **LD**, Lyme disease; **NB**, Neuroborreliosis; **OTC**, over the counter; **PCR**, polymerase chain reaction; **PPM**, permanent pacemaker; **PTLDS**, Post-treatment Lyme disease syndrome; **QALY**, Quality-adjusted life years; **TPW**, temporary pacing wire; **USA**, United States of America; **USD**, United States Dollar

Indirect costs that were incorporated for societal perspectives included: out-of-pocket drug costs, caregiving, travel, work loss, restricted-activity days at home, and loss of healthy time from sequelae. The study by Joss *et al*. incorporated the cost in the management of patients (e.g. consultations and screening test costs) found not to have evidence of the disease to evaluate non-confirmed LD patient burden from a societal perspective. [29] Two studies used a human capital approach,[30,36] one study used friction cost methods, [37] and three studies used secondary data to estimate indirect costs. [28,29,31]

#### Study findings

Three diagnostic cost analyses were included in this review (Table 4). Reported costs are standardized to 2017 USD currency using its respective inflation. The first US study by Strickland *et al*. concluded that 30,000 tests for LD were performed annually on Maryland residents, totalling direct medical costs of over 3.23M USD.[27] More recently, Hinckley *et al*. concluded that 3.4 million LD tests were conducted by the seven laboratories involved in their study (from four endemic states: Connecticut, Maryland, Minnesota and New York), at an estimated national cost of 566M USD.[34] Both studies concluded that diagnosis costs are a concern and should be included in the public health burden of LD. In Europe, a study by Muller *et al*. concluded that the overall expected cost of diagnostic testing and treatment was estimated at 67.93M USD in Germany, and suggested a high amount of potentially inappropriate healthcare services utilized for patients with a suspected or confirmed diagnosis of Lyme borreliosis.[32]

Overall, there were six cost analyses that assessed the economic burden of LD from a societal perspective: four from Europe, and two from the US (Table 4). Joss *et al*. reported an annual national economic burden of 735,550 USD (0.14 USD per capita, n = 5.40M) for Scotland,[29] while Henningsson *et al*. reported a national economic burden of 712,808 USD over 5 years in Sweden (0.07 USD per capita, n = 9.96M) for neuroborreliosis-related healthcare.[31] Furthermore, Lohr *et al*. reported an annual national economic impact of over 40.88M USD in Germany (0.51 USD per capita, n = 80.59M),[36] and van den Wijngaard *et al*. reported an annual national cost of 23.12M USD for LB in the Netherlands (1.36 USD per capita, n = 17.08M).[37]

In the US, cost analyses were completed sporadically from 1998 to 2015. A cost-of-illness study by Maes *et al*. reported an expected national expenditure of 3.93 billion USD over five years (2.41 USD per capita per year, n = 326.63M).[28] A healthcare utilization study by Zhang *et al*. reported direct medical costs of 4,273 USD and indirect costs of 7,484 USD per LD patient, totalling an estimated nationwide economic impact of 292M USD (0.89 USD per capita, n = 326.63M).[30] Similarly, a recent study by Adrion *et al*. reported an additional 3,084 USD of healthcare costs per LD patients over a 12-month period. They also determined that persistent LD sequelae (post-treatment LD syndrome, PTLDS) are associated with an increase of 3,946 USD healthcare costs compared to patients without PTLDS.[35]

## Discussion

We summarized a total of 21 economic evaluations (10 CEA and 11 cost analyses) related to LD. The majority of CEA studies were conducted prior to 2003, which was related to the previously available LD human vaccine. [9] Since the vaccine was withdrawn, there has been no novel intervention strategies for LD and subsequently minimal interest in CEA studies after 2003. Although this vaccine was withdrawn for reasons other than cost-effectiveness, [9] all four LD vaccination CEAs concluded that universal vaccination in the US was likely not cost-effective.

We included seven cost analyses focused on overall healthcare costs, three studies focused on diagnostic testing and one cost analysis focused on Lyme cardiac treatment. A common theme of the diagnostic cost analyses was the burden of inappropriate and over-usage of LD diagnostic testing in the US and Germany. While diagnostic economic evaluations specifically looking at costs are appreciated, it would be difficult for decision-makers to use this evidence in the absence of overall healthcare burden. Of the seven cost analyses assessing burden through total healthcare costs, three European studies concluded that further research and priority should be placed on preventive interventions for LD. Based on the most recent study by Zhang et al, the inflated annual economic impact for LD in the US was 292M USD. While this does not come close to the burden of influenza, cancer or chronic conditions (e.g. diabetes, obesity), it falls in the same magnitude of other high-profile vector-borne diseases in the US such as West Nile virus (778M USD over 13 years),[38] and Zika virus (500M USD annual assuming a 0.3% attack rate across six prominent states).[39] Overall, the economic burden of LD could be considered significant to the US and other developed countries to justify further research efforts in LD control and management.

There are limitations to this review, as resource constraints limited our literature search to articles written in English, introducing possible language bias. As a result, there may be an underrepresentation of European studies, which should not be interpreted as a lack of interest or lower LD incidence rates in this region. We did not attempt to identify costs associated with LD avoidance (i.e. non-health related prevention) since we were interested in the economic burden of LD on the healthcare system and society. Per capita costs were presented by dividing nation-wide burden by the entire population. However, it should be acknowledged that not everyone from a specific country are susceptible to LD. As a result, our review may be underestimating the actual per capita costs in high incidence areas, and we advise against using these per capita estimates to describe LD burden. Lastly, since the goal of this scoping review was to characterize the literature, risk of bias assessment and quality appraisal were not completed. We propose quality appraisal of the literature be explored in a future systematic review.

To our knowledge, this was the first study that systematically identified and characterized the economic evaluation literature for LD. In 1999 and 2002, reviews by Rouf *et al*. and Tella *et al*. identified costs, and cost-effectiveness studies in rheumatology, respectively.[40,41] However, both reviews identified limited LD studies and were not able to provide a comprehensive description of the burden of LD. The search strategy was comprehensively designed and adapted to four electronic databases to search NA and European literature. Given the amount of HTA and health economics organizations that release reports on vector-borne diseases, our search in the grey literature added to the comprehensiveness of this review. The timing of this review should be useful for health services and LD researchers alike aiming to understand the implications of this emerging infectious disease where it is estimated that 300,000 cases of LD are diagnosed annually in the US,[42] with limited development of novel interventions. [43]

A recent scoping review from Canada by Greig *et al*. identified all LD literature (e.g. risk factors, surveillance, diagnostics) related to public health. In this review, they identified 32 hits related to economic burden of LD or cost-benefit of interventions, but do not specifically report on the results, trends or conclusions of the studies. [44] In comparison, we included fewer studies since we excluded abstracts, editorials, secondary reviews, and economic evaluations not directly related to Lyme disease. Our review comes to a similar conclusion in that economic burden studies for LD are limited.

Our review was able to highlight specific research gaps in the LD literature. Of the 11 cost analyses, six studies reported societal costs (i.e. productivity loss, indirect costs, non-medical costs) between 23 and 64% of total economic costs. However, many of these indirect costs were roughly estimated using friction cost or human capital approach methods. It is evident that while healthcare costs are significant for LD in various countries, the societal costs are equally as impactful for this disease and should be further studied. Our review also summarized the range of economic impact across various countries known to have increasing rates of LD, and countries that have not estimated the economic impact of this vector-borne disease while facing increasing LD cases (e.g. Canada)[45] or historically have high LD incidence rates (e.g. Slovenia, Czech Republic).[46] Future efforts in identifying specific LD stages, indirect costs, or healthcare utilization that create the highest economic burden can be useful to support public health agenda in countries with this vector-borne disease.

There was a high degree of heterogeneity in economic evaluation methods, data sources and outcomes reported. The cost/QALY gained outcome is typically used to express the cost-effectiveness to health policy decision-makers, since it can be compared to commonly-used thresholds (e.g., $50,000/QALY,[47] and 20,000 Sterling Pounds/QALY in the United Kingdom [48]). However, many studies reported cost-effectiveness in other units, limiting appropriate comparisons. We also noticed an array of LD health states, and health state utility values (HSUV) used. HSUVs for LD health states were mostly derived from expert clinical opinion, which could in turn be underestimating the QALYs and the cost-effectiveness of interventions. Furthermore, an individualized approach (e.g. individual-level microsimulation) may be more accurate in predicting cost-effectiveness of LD interventions, since unique baseline characteristics of patients (e.g. comorbidities and demographics) can affect disease progression and subsequently predicted lifetime outcomes.

Only cost analyses from the US provided sequelae-attributable costs and case-attributable costs per patient.[35] As big data and computing power evolve in health care, future studies can further investigate attributable healthcare costs using health administrative data to determine population-specific burden. Future health services research should thus consider the local context in generating evidence to support health decision makers given the regional differences in LD incidence, detection, symptoms, sequelae and healthcare systems.

## Conclusions

This scoping review identified 21 economic evaluations for Lyme disease from North America and Europe. Similar to other vector-borne diseases, the burden of Lyme disease suggests an economic argument for further research. A greater understanding of the indirect costs of Lyme disease and cost-effectiveness of interventions in countries where the incidence rates of the disease are increasing, is warranted for guiding Lyme disease evidence-informed health policy decision making.

## Supporting information

S1 Text Medline search strategy (on November 08, 2017). (DOCX)

S1 Table PRISMA checklist. (DOC)
