# Supplementary material for: The economic burden of Lyme disease and the cost-effectiveness of Lyme disease interventions: A scoping review
Source: PLoS One. 2019 Jan 4;14(1):e0210280. doi: 10.1371/journal.pone.0210280 (PMC6319811; doi:10.1371/journal.pone.0210280)
Supplement: S1 Text — (DOCX) [file pone.0210280.s001.docx]

**S1 Text: Medline Search Strategy (on November 08, 2017)**

Database: Ovid MEDLINE: Epub Ahead of Print, In-Process & Other Non-Indexed Citations, Ovid MEDLINE® Daily and Ovid MEDLINE® <1946-Present>

Search Strategy:

1 Lyme Disease/ (9648)

2 Lyme Neuroborreliosis/ (715)

3 Erythema Chronicum Migrans/ (696)

4 Borrelia Infections/ or borrelia burgdorferi group/ or borrelia burgdorferi/ (8013)

5 (lyme or neuroborreliosis or borreliosis or "erythema migrans" or "erythema chronicum migrans" or ((borrelia or b) adj1 (afzelii or burgdorferi or garinii))).ab,ti,kw,kf. (14607)

6 ("persistent Lyme" or "chronic Lyme" or "post-Lyme" or "post-treatment Lyme disease" or PTLDS or PLDS).ab,ti,kw,kf. (1004)

7 1 or 2 or 3 or 4 or 5 or 6 (16641)

8 economics/ or economics, hospital/ or economics, medical/ or economics, nursing/ or economics, pharmaceutical/ or models, economic/ or health care sector/ (68096)

9 resource allocation/ or "cost allocation"/ or "costs and cost analysis"/ or cost-benefit analysis/ or "cost control"/ or "cost of illness"/ or "cost sharing"/ or health care costs/ or health expenditures/ (205753)

10 "deductibles and coinsurance"/ or Medical savings accounts/ or Direct service costs/ or Drug costs/ or Employer health costs/ or Hospital costs/ or Value of life/ or hospital charges/ or fees, medical/ or exp "fees and charges"/ or exp budgets/ (72212)

11 ((low adj2 cost*) or (high adj2 cost*) or (health?care adj2 cost*) or (fiscal or funding or financial or finance) or (cost adj2 estimate*) or (cost* adj2 variable) or (unit adj2 cost*) or cost* or (economic* or incremental* or cost*)).ab,ti,kw,kf. (833287)

12 8 or 9 or 10 or 11 (972973)

13 7 and 12 (255)
